# Poplar saplings exposed to recurring temperature shifts of different amplitude exhibit differences in leaf gas exchange and growth despite equal mean temperature

**DOI:** 10.1093/aobpla/plu018

**Published:** 2014-04-11

**Authors:** Sofia Cerasoli, Timothy Wertin, Mary Anne McGuire, Ana Rodrigues, Doug P. Aubrey, João Santos Pereira, Robert O. Teskey

**Affiliations:** 1 School of Agriculture, Forest Research Centre, Technical University of Lisbon, Lisboa, Portugal; 2 Warnell School of Forestry and Natural Resources, University of Georgia, Athens, GA, USA; 3 Institute for Genomic Biology, University of Illinois, Urbana, IL 61801, USA; 4 Department of Biology, Georgia Southern University, Statesboro, GA, USA

**Keywords:** *A* : *R* ratio, carbon allocation, leaf proteins, *Populus deltoides* × *nigra*.

## Abstract

Most investigations of plant responses to changes in temperature have focused on a constant increase in mean day/night temperature without considering how differences in temperature cycles can affect physiological processes and growth. To test the effects of changes in growth temperature on foliar carbon balance and plant growth, we repeatedly exposed poplar saplings (*Populus deltoides* × *nigra*) to temperature cycles consisting of 5 days of a moderate (M, +5 °C) or extreme (E, +10 °C) increase in temperature followed by 5 days of a moderate (M, −5 °C) or extreme (E, −10 °C) decrease in temperature, with respect to a control treatment (C, 23.4 °C). The temperature treatments had the same mean temperature over each warm and cool cycle and over the entire study. Our goal was to examine the influence of recurring temperature shifts on growth. Net photosynthesis (*A*) was relatively insensitive to changes in growth temperature (from 20 to 35 °C), suggesting a broad range of optimum temperature for photosynthesis. Leaf respiration (*R*) exhibited substantial acclimation to temperature, having nearly the same rate at 13 °C as at 33 °C. There was no evidence that preconditioning through temperature cycles affected the response of *A* or *R* to treatment temperature fluctuations. Averaged across the complete warm/cool temperature cycle, the *A* : *R* ratio did not differ among the temperature treatments. While foliar carbon balance was not affected, the temperature treatments significantly affected growth. Whole-plant biomass was 1.5 times greater in the M treatment relative to the C treatment. Carbon allocation was also affected with shoot volume and biomass greater in the M and E treatments than in the C treatment. Our findings indicate that temperature fluctuations can have important effects on growth, though there were few effects on leaf gas exchange, and can help explain differences in growth that are not correlated with mean growth temperature.

## Abstract (precis)

Most investigations of plant responses to changes in temperature have focused on a constant increase in temperature. However, changes in fluctuation in temperature, even if the mean temperature is the same, may affect plant growth. We tested the effects of weekly warm and then cool moderate (5°C) and large (10°C) fluctuation in temperature (with the same biweekly temperature sum) on plant growth. We found that, while the ratio of photosynthesis to respiration did not change, fluctuations in temperature did increase biomass accumulation and alter biomass allocation. Our findings suggest that, like mean temperature, fluctuation in temperature can significantly impact plant growth.

## Introduction

Along with increases in mean global temperature (Meehl *et al.* 2007), global climate models predict future increases in the frequency and magnitude of sudden episodes of extreme temperature (Coumou and Rahmstorf 2012). The increase in the frequency of heat waves observed in recent years (Meehl and Tebaldi 2004; Chase *et al.* 2006; Gershunov *et al.* 2009) exemplifies these expected temperature fluctuations. While there is a substantial body of information about the response of trees to changes in mean growth temperature (e.g. Sage and Kubien 2007; Way and Oren 2010), the impact of variable temperature patterns on plant growth has been largely unexplored. A short-term shift to a higher temperature caused a change in growth in herbaceous communities (De Boeck *et al.* 2011; Dreesen *et al.* 2012) and in foliar gas exchange of tree seedlings (Bolstad *et al.* 2003; Ameye *et al.* 2012). Bauweraerts *et al.* (2013) exposed *Quercus rubra* seedlings to repeated cycles of fluctuating temperatures, and observed significant decreases in gas exchange and total biomass due to the fluctuating temperatures.

However, most studies have traditionally compared plant responses to differences in mean temperature. In many cases, tree biomass accumulation was positively correlated with mean growth temperature (Usami *et al.* 2001; Allen and Vu 2009; Ghannoum *et al.* 2010; Way and Oren 2010; Duan *et al.* 2013), though this response was not universal and some experiments reported no increase in biomass accumulation when mean growth temperature was increased (De Lucia *et al.* 1994; Maherali and DeLucia 2000). The conflicting effect of changes in temperature on plant biomass may be due, in part, to the variable effect changes in temperatures may have on foliar carbon balance. The effect of temperature on growth will most likely be determined by a combination of factors, including the thermal sensitivity of growth (Atkin *et al.* 2006*a*), the temperature optimum for photosynthesis (Berry and Bjorkman 1980), the acclimation of respiration (Atkin *et al.* 2005) and, in some cases, the acclimation of photosynthesis (Ghannoum *et al.* 2010; Gunderson *et al.* 2010).

The objective of this study was to evaluate the impact of repeated cycles of shifting temperatures on growth and physiology, independent of changes in mean temperature. We repeatedly exposed *Populus deltoides* × *nigra* saplings to temperature cycle treatments consisting of a 5-day period of a moderate (M, 5 °C) or extreme (E, 10 °C) temperature increase followed by a 5-day period of a moderate (M, 5 °C) or extreme (E, 10 °C) temperature decrease relative to a non-cycling control (C) treatment and compared volume growth, shoot and root biomass and leaf gas exchange. Plants in all three treatments were exposed to the same mean temperature over each cycle of warm and cool periods and over the entire growth period. In addition, a subset of control plants were exposed only once to the extreme temperature treatment to determine whether their physiological response would differ from that of plants repeatedly exposed. We tested the hypotheses that (i) growth will be reduced in the M and E temperature treatments compared with C; (ii) *A* will be affected much more than *R* by changes in temperature; and (iii) prior exposure to the warm and cool periods will alter the rates of *A* or *R* when plants are subsequently exposed to those conditions compared with plants not previously exposed.

## Methods

### Plant material and experimental design

*Populus deltoides* × *nigra* cuttings (OP-36; Segal Ranch Hybrid Poplars, Grandview, WA, USA) were planted in 8-L pots in a potting medium (Nursery Mix; Conrad Fafard Inc., Agawam, MA, USA). Plants were grown in environment-controlled chambers (Model GC36; EGC Inc., Chagrin Falls, OH, USA) at The University of Georgia in Athens, GA, USA. The temperature (15 °C), relative humidity (70 %) and photosynthetic active radiation (PAR, 300 µmol m^−2^ s^−1^) under a 12-h day/12-h night period were constant prior to treatment implementation.

**Figure 1.**
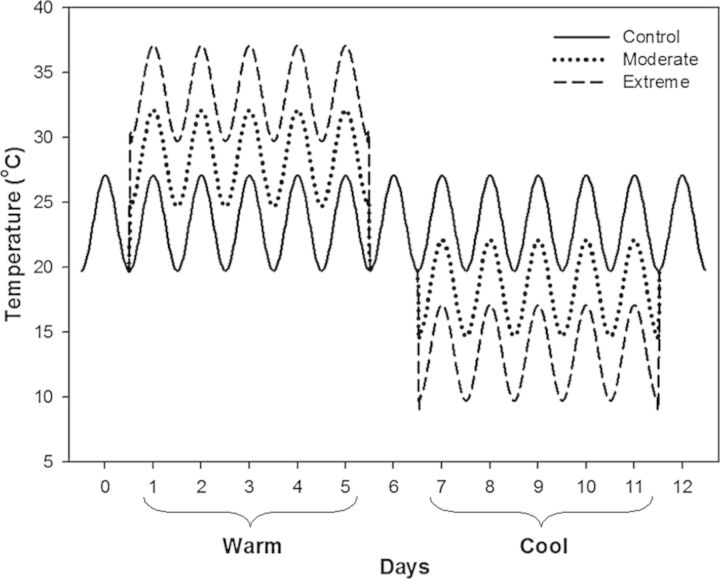
Air temperature (°C) regimes imposed during a complete cycle of 5-day warm and 5-day cool periods in the control treatment as well as the moderate (M) and extreme (E) temperature cycle treatments.

After 1 month under pre-treatment conditions, the average stem height and diameter at the root collar were 9.9 cm (±0.87) and 2.6 mm (±0.09), respectively. Plants were supplied with 16.5 g of 15-9-12 (NPK) time-release fertilizer (Osmocote; Scots Co., Marysville, OH, USA) and randomly divided among three environment-controlled chambers. Each chamber was assigned a different temperature treatment: control (no temperature shifts) (C), moderate temperature shifts (M) and extreme temperature shifts (E). The C treatment consisted of constant diel variation with a minimum night temperature of 19.7 °C and a maximum day temperature of 27.0 °C (Fig. 1). The temperature shift treatments consisted of a 5-day period of a day/night temperature increase of 5 °C (M) or 10 °C (E) (hereafter referred to as the warm period) followed by a similar 5-day period of a day/night temperature decrease of 5 °C (M) or 10 °C (E) (hereafter referred to as the cool period). At the end of each warm and cool period, temperature shift treatments returned to C treatment temperature for 1 day. Thus, a complete temperature cycle of one warm and one cool period lasted 12 days. Day length (12 h), PAR measured at the top of the canopy (300 µmol m^−2^ s^−1^), relative humidity (50 %) and diel temperature variation (7.3 °C) were identical among treatments. The mean daily temperature across the 12-day cycle was the same (23.4 °C) among all treatments. The entire temperature cycle of warm and cool periods was repeated four times. Temperature was monitored inside the chambers with thermocouples located just above the canopy. Pots were elevated 10 cm above the chamber floor to improve air circulation. Plants were rotated and treatments were reassigned among the chambers every 6 days.

At the beginning of the experiment, 22 plants were assigned to the C treatment, and 12 plants each were assigned to M and E treatments. To determine whether prior temperature conditions influenced the rates of *A* or *R*, a single-exposure temperature shift (S) treatment was also implemented, consisting of three plants from the C treatment that were transferred to the E treatment at the beginning of the third warm period and another three plants transferred to the E treatment at the beginning of the third cool period.

### Physiological measurements

Leaf gas exchange was measured during the third temperature cycle on plants in the three treatments (C, M and E + S). Net assimilation (*A*) and leaf dark respiration (*R*) were measured with a portable photosynthesis system in open path configuration (LI6400; Li-Cor, Lincoln, NE, USA) equipped with a blue–red LED light source (LI6400-02B) and a CO_2_ injection system (LI6400-01). Net assimilation (*A*) was measured on one leaf per plant between 1000 and 1200 h on the first, third and fifth day of the warm and cool periods (which correspond to days 1, 3, 5, 7, 9 and 11 of the temperature cycle; Fig. 1). Measurements were conducted at chamber conditions (relative humidity: 50 %; [CO_2_]: 450 µmol mol^−1^; PAR: 300 µmol m^−2^ s^−1^) and cuvette temperature was set to approximate air temperature in each chamber (i.e. 25.4, 30.4 and 35.4 °C during the warm period and 25.4, 20.4 and 15.4 °C during the cool period for C, M and E + S, respectively). Leaf *R* measurements were made in the dark using a similar protocol between 2000 and 2200 h on the same days. Cuvette temperature was set at 23.4, 28.4 and 33.4 °C during the warm period and 23.4, 18.4 and 13.4 °C during the cool period for C, M and E + S treatments, respectively.

Measurements were repeated on five individual plants in each of the C, M and E treatments across the 12-day cycle. Repeated measurements were conducted on the same single leaf per plant over the entire cycle. For the S treatment, measurements were repeated on three plants during the warm period and three different plants during the cool period.

The relationship between *A* and leaf internal CO_2_ concentration (*A*/*C*_i_) was measured on four plants per treatment at the end of both the warm and cool periods of the third cycle. For these measurements, plants were moved to a different growth chamber with similar light intensity and relative humidity of the treatment chambers, but temperature was maintained at 25 °C. Cuvette conditions were set to mimic chamber conditions, with the exception that measurements were made at saturating PAR (1200 µmol m^−2^ s^−1^). *A*/*C*_i_ curves started at 400 µmol mol^−1^ CO_2_ and ranged from 40 to 1000 µmol mol^−1^ CO_2_ in nine steps. The maximum rate of carboxylation (*V_c_*_max_) and photosynthetic electron transport (*J*_max_) were determined with the A/*C*_i_ Curve Fitting Utility version 0.4 (Sharkey *et al.* 2007).

### Growth measurements

Stem height and root collar diameter of each plant were measured at the end of each warm and cool period. Stem volume was calculated as the product of stem height and stem area at base. No branches developed on the stems during the study. Plants were harvested at the end of the fourth temperature cycle. The total leaf area of each plant was measured with a leaf area meter (LI 3100; Li-Cor Inc.). Biomass was separated into leaves, stem and roots, dried to constant mass at 70 °C, and weighed. Leaf area was divided by leaf weight to determine the specific leaf area (SLA) for each plant.

### Data analysis and statistics

One-way ANOVA was used to test mean treatment differences in *A*, *R*, *g*_s_ and *A* : *R* for each temperature period as well as to test treatment differences in *V_c_*_max_ and *J*_max_. The influence of prior exposure to recurring temperature cycles was tested by comparing means of the E and S treatments. A repeated-measures design was used to test treatment differences in stem volume throughout the observation period. Temperature treatment (C, M and E) was a fixed factor, day (*n* = 9) was the fixed repeated factor and individual plant (*n* = 12 for M and E; *n* = 16 for C) was the random subject factor. Differences in relative growth rate, calculated as (ln(mass2) − ln(mass1))/(DOY2 − DOY1), among and within treatments between the warm and cool period were tested by ANOVA. Biomass, total leaf area and SLA were analysed using a one-way ANOVA. Temperature treatment (C, M and E) was a fixed factor and individual plant (*n* = 12 for M and E; *n* = 16 for C) was a random factor. Linear and non-linear regression were used to plot the response of *R*, *g*_s_ and *A*, respectively, to leaf temperature. All analyses were performed using SAS (Version 9.1.3; SAS Inc., Cary, NC, USA) with a type-1 error rate of 0.05. Treatment means were compared using Fisher's least significant difference (LSD) test.

## Results

### Leaf gas exchange

**Figure 2.**
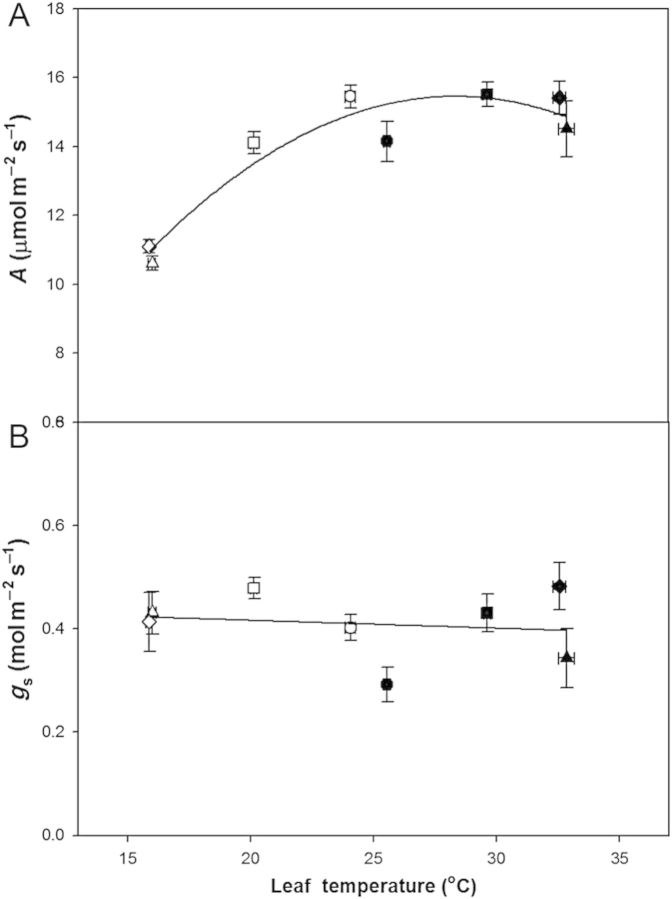
Mean (A) leaf net photosynthesis (*A*) and (B) stomatal conductance (*g*_s_) of poplar saplings measured at the mean temperature of the daily light period during a 5-day warm period (filled symbols) and a 5-day cool period (open symbols) for the C (circle), M (square), E (triangle) and S (diamond) treatments. Measurements of *A* and *g*_s_ were made on days 1, 3 and 5 of each warm and cool period and averaged across the period. Error bars are ±1 SEM. The curve represents best-fit non-linear regression (*R*^2^ = 0.84).

In both the warm and cool periods, *A* and *R* were measured at the mean temperature of the light or dark period of each treatment. The pattern of response of *A* across the C, M and E treatments in both the warm and cool periods indicated a broad temperature optimum for *A*, which produced very similar values of *A* across a range of measured temperatures from 20 to 35 °C (Fig. 2A; *P* = 0.4 linear regression analysis). For example, regardless of warm or cool period, mean *A* measured in the E treatment during the warm period (leaf temperature of 32.8 °C) was similar to *A* measured in the M treatment during the cool period (leaf temperature of 20.1 °C) (14.4 vs. 14.1 μmol m^−2^ s^−1^, respectively; *P* = 0.38). However, it should be noted that *A* was significantly lower at the lowest measurement temperature (E treatment in the cool period; leaf temperature of 15.9 °C) with a mean *A* of only 11.1 μmol m^−2^ s^−1^, compared with the next lowest measurement temperature (M treatment in the cool period: 20.4 °C; *P* < 0.001). This low-temperature reduction in *A* was the only treatment effect: *A* was significantly different in the E treatment only during the cool period compared with the M and C treatments (*P* < 0.001 for both). There were no significant differences among the C, M or E treatments during the warm period, or between the C and M treatment in the cool period (*P* > 0.21 for all comparisons). There was no effect of measurement temperature on *g*_s_ (Fig. 2B; *P* = 0.37 for linear regression).

There was also no apparent effect of repeated temperature cycles on the response of *A* to the different treatment temperatures. Plants that were moved from the C treatment to the E treatment on the first day of the warm period and on the first day of the cool period (S treatment) did not have significantly different *A* when compared with plants that had been subjected to two previous complete cycles of warm and cool periods (compare E and S, Fig. 2A) (warm period: *P* = 0.63; cool period: *P* = 0.63).

**Figure 3.**
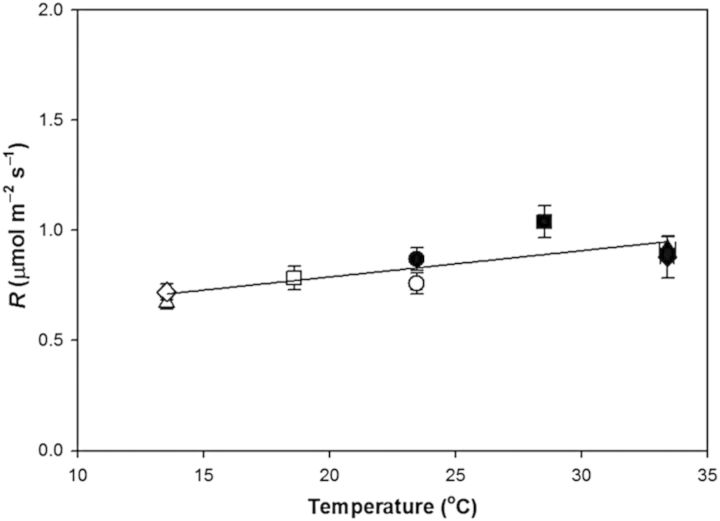
Mean leaf dark respiration (*R*) of poplar saplings measured at the mean temperature of the daily dark period during a 5-day warm period (filled symbols) and a 5-day cool period (open symbols) for the C (circle), M (square), E (triangle) and S (diamond) treatments. Measurements of *R* were made on days 1, 3 and 5 of each warm and cool period and averaged across the period. Error bars are ±1 SEM. The line represents linear regression.

The response of *R* to the treatments also displayed little sensitivity to temperature (Fig. 3). During the warm period, *R* was not significantly different between the C, M or E treatments (*P* = 0.16). Of note, in the warm period, *R* in the E treatment (measured at 33.4 °C) was actually slightly lower than *R* in the C treatment (measured at 23.5 °C), but the difference was not significant (−8.6 %; *P* = 0.32). Similarly, in the cool period, *R* was not affected by the treatments (*P* = 0.11); *R* in the E treatment (measured at 13.5 °C) was nearly identical to *R* in the C treatment (measured at 23.5 °C) (−0.794 vs. −0.796 µmol m^−2^ s^−1^; *P* = 0.97). While the lack of treatment effects within the warm and cool treatment periods highlights the plasticity of *R*, there were some limitations in its capacity for acclimation. When compared between the warm and cool period in the E treatment (a 20 °C difference in measurement temperature), *R* was significantly different (−0.92 vs. −0.67 μmol m^−2^ s^−1^; *P* = 0.001). However, when *R* was plotted against measurement temperature (Fig. 3), the slope of the relationship, while significantly different from zero, was quite small (*R* = −0.0146 × Temp−0.485; *P* = 0.012) and *Q*_10_ was dramatically smaller (0.015) than the assumed value of 2 in the absence of acclimation.

The plants in the E and S treatments had nearly identical responses of *R* to temperature (warm period: *P* = 0.43; cool period: *P* = 0.70), suggesting that substantial temperature acclimation of *R* was not the result of preconditioning in previous temperature cycles.

**Figure 4.**
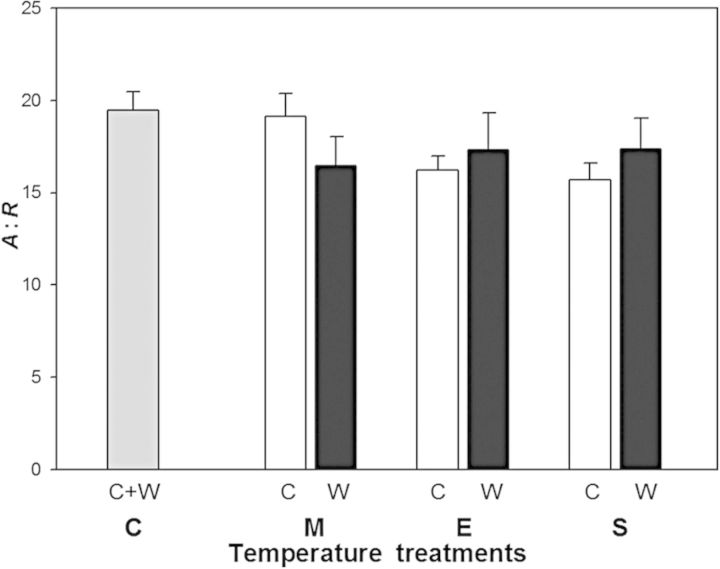
The ratio of net photosynthesis to respiration (*A* : *R*) of leaves. The control (C), moderate (M), extreme (E) and shift from C to E (S) treatments are indicated by letters in bold. Filled bars represent measurements made during the warm period (W), and open bars are measurements during the cool period (C) for the M, E and S treatments. The control treatment (C) bar is the average of both periods. Error bars are ±1 SEM.

Mean *A* : *R* was similar for the C, M and E treatments (Fig. 4) (*P* = 0.17). Averaged across both the warm and cool periods, it ranged from 19.5 in the C treatment to 16.8 in the E treatment, a non-statistically significant difference (*P* = 0.063). Within the M and E treatments, there were no statistically significant differences in mean *A* : *R* when compared between the warm and cool periods (*P* = 0.58 and *P* = 0.18, respectively). That observation was also consistent in the S treatment which had an *A* : *R* of 16.6 (compared with 16.8 in the E treatment; *P* = 0.87), providing further evidence that the temperature responses of *A* and *R* observed in this study did not result from temperature preconditioning.

The C, M and E treatments did not influence *V_c_*_max_ (*P* = 0.92) or *J*_max_ (*P* = 0.81) measured at the end of the warm or cool period (i.e. no temperature treatment × period interaction; *P* = 0.053 and *P* = 0.38, respectively). Further, *V_c_*_max_ and *J*_max_ remained similar between the warm and cool periods (*P* = 0.36 and *P* = 0.55, respectively). Averaged across treatments and periods, *V_c_*_max_ was 74.58 ± 1.80 and *J*_max_ was 116.88 ± 3.02. *V_c_*_max_ and *J*_max_ were also similar in the E and S treatments at the end of both the warm (*P* = 0.83 and *P* = 0.84, respectively) and cool (*P* = 0.35 and *P* = 0.93, respectively) periods.

### Growth and leaf area

**Figure 5.**
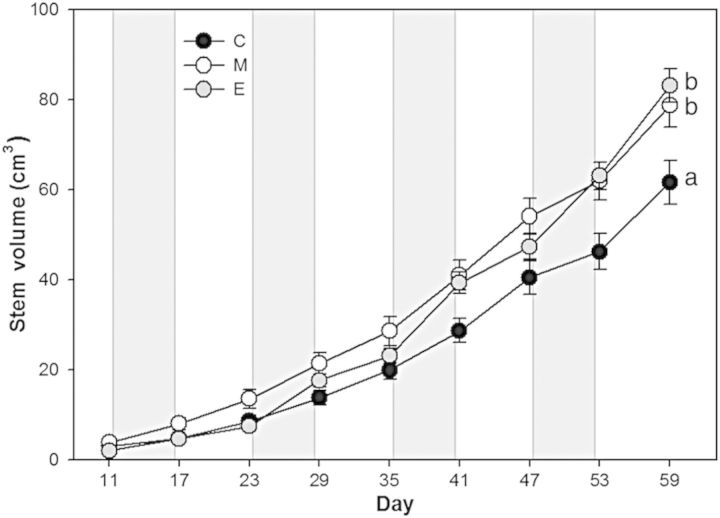
Mean stem volume [height × PI(diameter/2)^2^] estimates of poplar saplings over the four temperature cycles during the experiment. Treatments are control (C), moderate (M) and extreme (E) temperature cycles. Warm periods are depicted by grey bars. Different lowercase letters indicate significantly different final stem volume (Fisher's LSD, *α* = 0.05). Error bars are ±1 SEM.

Temperature treatments had a significant effect on stem height and stem volume, and the effect increased as the experiment progressed (Fig. 5). Treatment effects on stem volume became apparent by the end of the third warm period (day 41). By the end of the fourth temperature cycle (day 59), stem volume was significantly greater in the M treatment (82.95 ± 7.85 cm^3^) and the E treatment (82.79 ± 5.38 cm^3^) than in the C treatment (57.78 ± 6.34 cm^3^). At the end of the experiment, plants in the E treatment were significantly taller than plants in either the M or C treatment (84 vs. 76 cm (*P* = 0.5) or 67 cm (*P* < 0.001), respectively), which were also significantly different from each other (*P* = 0.03). Leaf area per plant was not significantly different among treatments, although there was a trend of greater leaf area in the M and E treatments compared with the C treatment (Table 1). Leaf dry mass per plant was significantly higher in both the M and E treatments than in the C treatment. These differences resulted in lower SLA in the M and E treatments than in the C treatment.

**Table 1.** Mean ± SE leaf area, leaf dry mass and SLA of poplar saplings harvested at the conclusion of four recurring temperature cycles consisting of 5-day warm and 5-day cool periods with temperature shifts of 5 °C (M) or 10 °C (E) above and below control (C). Treatment means with different lowercase letters are significantly different (Fisher's LSD, *α* = 0.05). Bold font indicates significant *P*-values.

Treatment	C	M	E	*P*-value
Leaf area (m^2^)	0.23 ± 0.02	0.28 ± 0.02	0.27 ± 0.01	0.0662
Leaf dry mass (g)	10.92 ± 0.90^b^	15.74 ± 1.24^a^	14.99 ± 0.71^a^	**0**.**0016**
SLA (m^2^ kg^−1^)	21.74 ± 0.53^a^	18.01 ± 0.54^b^	18.19 ± 0.23^b^	**0**.**0001**

**Figure 6.**
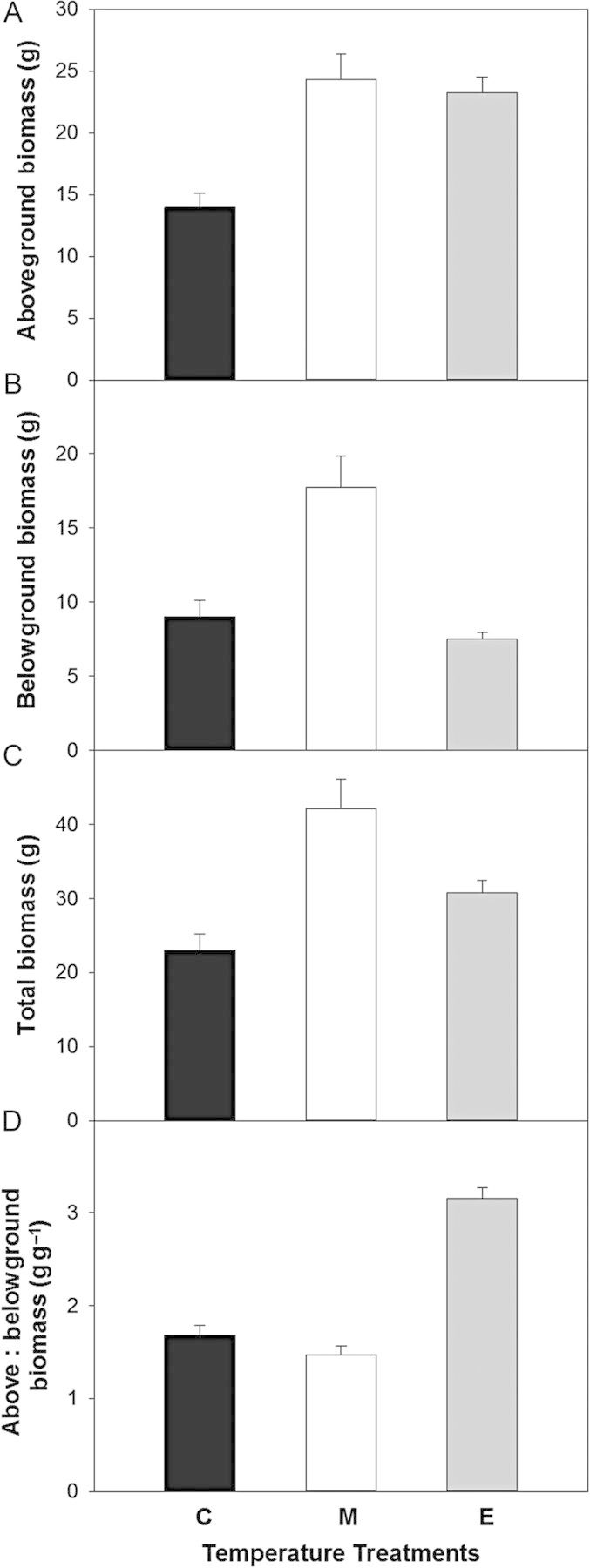
Mean aboveground biomass (A), belowground biomass (B), total biomass (C), and above : belowground biomass (D) of poplar saplings harvested at the end of the fourth temperature cycle. Treatments are control (C—black bars), moderate (M—white bars) and extreme (E—grey bars) temperature treatments. Error bars are ±1 SEM.

The M and E treatments increased aboveground biomass compared with the C treatment at final harvest, but belowground biomass responded differently (Fig. 6). Aboveground biomass was about 1.5 times greater in the M and E treatments than in the C treatment (Fig. 6A; *P* = 0.0020), while belowground biomass was greater in the M treatment than in the C and E treatments (Fig. 6B; *P* = 0.0005). As a result, total biomass was greater in the M treatment than in the C and E treatments (Fig. 6C; *P* = 0.0081), while the above- to belowground biomass ratio was about two times greater in the E treatment than in the C and M treatments (Fig. 6D; *P* = 0.0001). Relative growth rate (RGR) did not differ between the warm and cool periods in the C treatment (0.101 vs. 0.108; *P* = 0.4). However, in the E treatment RGR was nearly double in the cool periods compared with the warm periods (0.139 vs. 0.076; *P* < 0.001). In the M treatment RGR tended to be greater in the cool periods compared with the warm periods, though the difference was not significant (0.106 vs. 0.088; *P* = 0.08). Thus, in the E treatment, RGR was significantly greater in the cool periods compared with the C treatment (*P* < 0.001), but significantly lower in the warm periods compared with the C treatment (*P* < 0.001). There was no difference in RGR between the C and M treatments in either the warm or cool periods (*P* = 0.8 and *P* = 0.6, respectively).

## Discussion

One of the most important findings of this study was that growth was strongly affected by the temperature pattern to which the plants had been exposed. Growth did not simply correspond to the mean temperature or heat sum that the plants experienced during the growth period, since these were equal in the C, M and E treatments. Although we predicted that growth would be reduced in the M and E treatments relative to the C treatment (Hypothesis 1), those treatments actually increased growth. Of interest, the increase in biomass with fluctuating temperatures we report here was also accompanied by shifts in allocation. For instance, the E treatment caused a doubling in the root : shoot ratio, suggesting that fluctuations in temperature may alter growth rates and also affect biomass allocation patterns. These findings are especially relevant given the fact that fluctuations in ambient air temperatures in the past 20 years have become larger and have affected a greater land area (Hansen *et al.* 2012). This suggests that broad correlations between biomass and mean temperature may be insufficient to accurately estimate plant growth.

The response of *A* to temperature indicated a broad range of optimum temperature for *A* that was exceeded only at the lowest measurement temperature in the E treatment. This result provided little support for our second hypothesis that *A* would be more affected by temperature than *R*. Additionally, there was no difference among the C, M or E treatments in *V_c_*_max_ or *J*_max_ measured at a common temperature, indicating that the treatments caused no permanent differences in photosynthetic capacity.

In the same hybrid poplar genotype used in this study, Ow *et al.* (2008) found no evidence of acclimation of *A* to different growth temperatures, which was consistent with the response we observed. A lack of acclimation of *A* to a change in growth temperature has been reported in other tree species (e.g. Wertin *et al.* 2011), including other *Populus* species (Centritto *et al.* 2011; Dillaway and Kruger 2011). However, there are also tree species that do exhibit thermal acclimation of *A* (Ghannoum *et al.* 2010; Gunderson *et al.* 2010).

We observed acclimation of *R* to temperature, which was consistent with the results of Ow *et al.* (2008) in the same poplar genotype. By definition, full acclimation to temperature occurs when similar *R* is achieved under different temperature conditions, i.e. thermal homeostasis (Atkin *et al.* 2006*b*). In our study there were only small differences in *R* among treatments, indicating a substantial acclimation response, and near-thermal homeostasis across the wide range of measurement temperatures, from 33.4 °C in the warm period to 13.4 °C in the cool period in the E treatment.

Even though there were ±5 and ±10 °C differences in temperature in the M and E treatments compared with C, the *A* to *R* ratio did not differ among the three treatments. This indicates that average foliar carbon balance was not significantly affected by the large fluctuations in temperature that were imposed in this study. Atkin *et al.* (2006*b*) reported that *A* : *R* for *Plantago major* and *Plantago lanceolata* was similar across a 20 °C range of temperature. In a series of temperature manipulations using various grasses and trees, including *Eucalyptus delegatensis* and *E. dumosa*, Loveys *et al.* (2003) reported a strong correspondence between *A* and *R* across a variety of growth temperatures, which they attributed to acclimation of both *A* and *R*. Likewise, Gifford (1995) reported that *A* : *R* was extremely stable across a large range of temperatures if the plants were given adequate time to acclimate to the new growth temperature. However, in other studies *A* : *R* was sensitive to growth temperature. In *Populus euramericana*, *A* : *R* was greater in plants grown at 25 °C than at 35 °C, due mainly to a temperature-driven decline in *A* (Fares *et al.* 2011). In contrast, *A* : *R* increased in *Populus balsamifera* with an increase in growth temperature, due mainly to the insensitivity of *A* to an increase in growth temperature (Silim *et al.* 2010). While our findings support the conservation of *A* : *R* at different growth temperatures, this result could be influenced by the range of temperatures that the plants experience, and higher or lower temperatures beyond what were used in this study could alter the *A* : *R* ratio.

Another interesting finding was the decoupling of biomass production and *A* : *R*. The similarity of *A* : *R* among treatments suggests that final biomass should be similar as well. However, both stem volume growth measured throughout the study and total accumulated biomass measured at the end of the study differed substantially among the treatments, and were greater in the M and E treatments compared with the C treatment. This result suggests different temperature sensitivities of biomass accumulation and leaf gas exchange. It is possible that the optimum temperature for growth is different from the optimum temperature for *A*, and plants in the M and E treatments may have been exposed to temperatures more ideal for biomass accumulation compared with plants in the C treatment. Calculations of RGR results offer some support for this idea. RGR in both the M and E treatments was greater in the cool period compared with the warm period. This increase in RGR during the cool period may be due to a decrease in whole-plant respiration, resulting in more carbohydrates available for biomass accumulation, or a shift to temperatures more favourable for biomass accumulation. Regardless, our results suggest that fluctuating temperatures can promote whole-plant growth. However, the exact mechanism that caused the increase in growth with temperature fluctuations is not known.

Our results also indicate that carbon allocation in *P. deltoides* × *nigra* was sensitive to temperature. Sensitivity of carbon allocation to temperature appears to be present in some tree species but not in others. Pumpanen *et al.* (2012) found a lower root : shoot ratio in *Betula pendula* seedlings under low compared with high soil temperature, but no differences in *Pinus sylvestris* or *Picea abies.* An increase in temperature increased the root : shoot ratio in *Pinus tabulaeformis* but not in *Picea asperata* (Zhao and Liu 2009), while other studies reported increases in seedling growth with increasing temperature but no effect on the root : shoot ratio in *Pinus ponderosa* (De Lucia *et al.* 1994), *Fagus sylvatica* (Overdieck *et al.* 2007) and *P. asperata* (Zhao and Liu 2009).

One final question we addressed was whether prior exposure to the temperature cycles would change the response of *A* or *R* to temperature (Hypothesis 3). At the time we made gas exchange measurements, the plants in the M and E treatments had been previously exposed to two full temperature cycles. We found no evidence that *R* responded differently in these plants compared with plants that had no prior exposure to temperature fluctuations (S treatment). This finding is in contrast with that of Ow *et al*. (2008) in the same poplar clone, who found that full acclimation of *R* was observed only in leaves that emerged in the new temperature treatment. The reasons for differences in acclimation responses to temperature between these two studies are unclear. The question of whether the capacity for temperature acclimation of *R* in this species is an inherent characteristic of mature leaves or is dependent on the temperature exposure during leaf growth remains unresolved. We also found that *A* did not differ significantly between the E and S treatments, indicating that it was not altered by temperature acclimation, or stress, after exposure to repeated temperature cycles, compared with plants that had never been exposed to temperature shifts.

## Conclusions

In this study, volume and biomass growth increased in response to repeated cycles of fluctuating temperatures, even though there was little change in leaf respiration and net photosynthesis. The amplitude of the temperature shifts played an important role in the growth response of the plants. The greatest growth occurred in the moderate temperature treatment (M, ±5 °C). Aboveground growth increased in plants subjected to the extreme temperature treatment (E, ±10 °C), but not belowground growth or total biomass. The predicted response of trees to expected future changes in temperature is generally based on how they respond to increases in mean temperature without consideration of the possible effects of fluctuating temperatures or the magnitude of extreme events. Our results suggest that in addition to the influence of mean growth temperature, the amplitude of temperature shifts can have an important effect on growth, and this effect may not be predictable from measurements of leaf respiration and net photosynthesis.

## Sources of Funding

This work was supported by a grant from the United States Department of Energy NICCR Program (Grant: 07-SC-NICCR-1060). S.C. was supported by a Post-Doctoral Fellowship from Fundação para a Ciencia e Tecnologia (SFRH/BPD/28384/2006).

## Contributions by the Authors

S.C., T.W., M.A.M. and R.O.T. contributed to experimental design, measurements, data analysis and manuscript preparation. A.R. and J.S.P. contributed to data analysis. D.P.A. contributed to data analysis and manuscript preparation.

## Conflicts of Interest Statement

None declared.
